# Oral Manifestations of COVID-19: A Cross-Sectional Study of Their Prevalence and Association with Disease Severity

**DOI:** 10.3390/jcm11154461

**Published:** 2022-07-30

**Authors:** Nada O. Binmadi, Suad Aljohani, Maha T. Alsharif, Soulafa A. Almazrooa, Amal M. Sindi

**Affiliations:** Department of Oral Diagnostic Sciences, King Abdulaziz University Faculty of Dentistry, Jeddah 21589, Saudi Arabia; sraljohani@kau.edu.sa (S.A.); mtyalsharif@kau.edu.sa (M.T.A.); salmazrooa@kau.edu.sa (S.A.A.); amsindi@kau.edu.sa (A.M.S.)

**Keywords:** COVID-19, cross-sectional study, oral manifestations, oral cavity, SARS-CoV-2, taste disorders, xerostomia

## Abstract

Background: COVID-19, caused by SARS-CoV-2, has impacted the world in an unprecedented way since December 2019. SARS-CoV-2 was found in the saliva of patients, and entry points for the virus may have been through the numerous angiotensin-converting enzyme 2 receptors in the oral cavity. Oral manifestations of COVID-19 could contribute to the burden of oral disease. Objective: To determine the prevalence of oral manifestations of COVID-19 in patients and their association with disease severity. Methods: Interviews were conducted with adult participants diagnosed with COVID-19 between October 2021 and March 2022 to document their demographic and health status data, symptoms, and the presence of oral manifestations of COVID-19. Chi-square and the Fisher’s exact test were used to compare data on the presence or absence of oral manifestations of COVID-19. Results: Of 195 participants interviewed, 33% were 18 to 24 years old, 33% were 25 to 34 years old, and 75% were female. A total of 57 (29%) had oral manifestations; the most common were taste disorders (60%), xerostomia (42%), and oral ulcers (11%). There was no relationship between the severity of COVID-19 and the presence of the oral manifestations. Conclusion: Oral manifestations of COVID-19 were common among female patients and linked to certain general COVID-19 symptoms regarding frequency and extent.

## 1. Introduction

In December 2019 in Wuhan, Hubei Province, China, a cluster of patients were seen who had shortness of breath, fever, and pneumonia. On 7 January 2020, the Chinese government identified the cause of this pneumonia as a newly isolated coronavirus of 2019 (and the disease was named COVID-19). The virus quickly spread, and by 11 March 2020, more than 136 countries were affected by the disease. The World Health Organization (WHO) then declared the disease a global pandemic [1]. By 31 May 2022, COVID-19 had infected 528,816,317 individuals and resulted in 6,294,969 deaths globally [2]. Saudi Arabia reported its first case in March of 2020, and by 31 May 2022, the country had reported 768,648 cases of and 9149 deaths from the disease [3].

SARS-CoV-2, the agent of COVID-19, is a member of the family Coronaviridae. It belongs to the Betacoronavirus subfamily, together with two other highly pathogenic viruses, SARS-CoV and MERS-CoV. It is a positive-sense single-stranded RNA (+ssRNA) virus with envelope-anchored spike protein receptors that facilitate its entry into host cells [4,5]. Angiotensin-converting enzyme 2 (ACE2) is the main receptor involved in COVID-19 pathogenesis [5]. It is abundantly present on the ciliated cells of the airway epithelium, or alveolar type 2 cells, which are the primary sites attacked by the virus. Direct contact with patients and their respiratory droplets is a well-known mode of transmission [4].

The most common symptoms reported by patients with COVID-19 are fever; respiratory symptoms, such as a cough or shortness of breath; and general fatigue. Other less common symptoms include headache, taste distortion, anosmia, and sore throat. Additionally, some patients present with gastrointestinal symptoms, such as diarrhea, nausea, or vomiting. The severity of these symptoms depends on many factors, such as the time of exposure to the virus and the patient’s age and gender, as well as the presence of coexisting diseases. Investigators found that patients with autoimmune diseases were more prone to the infection [6].

At the beginning of the pandemic, it was assumed that the disease did not affect the oral cavity, and this was considered a factor that distinguished COVID-19 from other viral exanthemas. Later, however, SARS-CoV-2 was detected in the saliva of patients with COVID-19. The reverse transcriptase–polymerase chain reaction (RT-PCR) test of saliva was found to be more sensitive than a routine nasopharyngeal test in detecting the virus [6]. ACE2 receptors are distributed all over the oral cavity and especially on the dorsum of the tongue and in the salivary glands; these locations make these sites potential entry points for the virus. Therefore, COVID-19 could contribute to the burden of oral disease [6,7,8]. The most commonly reported affected sites were the tongue (38%), labial mucosa (26%), and palate (22%). Oral lesions were symptomatic in 68% of the cases [6,8,9].

Many researchers reported that oral manifestations were associated with COVID-19. The types of manifestations varied significantly. Taste disorder was the first and most common oral symptom [8]. Other oral manifestations were aphthous-like lesions, herpetiform lesions, periodontitis, candidiasis, mucormycosis and oral lesions of Kawasaki-like disease, pustules, a fissured or depapillated tongue, macules, papules, plaques, abnormal pigmentation, halitosis, whitish areas, hemorrhagic crusts, necrosis, petechiae, swelling, erythema, and spontaneous bleeding [5,7,9,10]. The risk of some of these manifestations can be reduced by taking probiotics, as proposed by Butera et al. [11]. Little is known about the prevalence of these oral manifestations in adults. It also has not been affirmed whether the oral symptoms are a direct manifestation of the infection or merely a consequence of the immune response to it. We aimed in the present study to determine the prevalence of the oral manifestations of COVID-19 in patients and their association with disease severity and general symptoms.

## 2. Materials and Methods

### 2.1. Study Design and Setting

A cross-sectional survey was carried out at the King Abdulaziz University Faculty of Dentistry (KAUFD), Department of Oral Diagnostic Sciences, Jeddah, Saudi Arabia, between October of 2021 and March of 2022 to determine the prevalence of oral manifestations in patients diagnosed with COVID-19 and their association with disease severity and to describe the patterns of the oral manifestations. Ethical approval to conduct the study was granted by the Research Ethics Committee at KAUFD (study approval number: 204-01-21). Potential participants were informed that their participation was voluntary and that they were free to decline to participate in the study or to halt participation at any point during the study. All participants provided written informed consent. All data were anonymized and stored on secure servers that could only be accessed by authorized healthcare professionals from the Department of Oral Diagnostic Sciences, Faculty of Dentistry, King Abdulaziz University.

### 2.2. Study Population

Participants were 18 years of age or older, had been diagnosed with COVID-19 between March of 2020 and March of 2022, could read and write in English/Arabic, and could provide informed consent. We included participants who stated that they had confirmed COVID-19 disease based on a test performed at a government center or a private hospital/laboratory or on a self-test [12].

### 2.3. Sample Size Calculation

We used the sample size for cross-sectional studies [13], in which published systematic reviews reported a prevalence of 45% of taste disorders in COVID-19 patients (95% confidence interval [CI], 34% to 55%) (taste disorders were the commonest oral manifestation of COVID-19) [14]. We used a *z*-value from the standard normal distribution that reflected a 95% CI (e.g., *z* = 1.96 for 95%) and a margin of error of 7% [15]. This yielded a sample size of 185. A random sampling technique was used to recruit participants.

### 2.4. Study Procedures

Invitations were sent randomly via email, WhatsApp, or SMS to recruit eligible candidates. A reminder was sent 2 weeks and 1 month after the first invitation was sent. If we received an acceptance, we invited the participant to an interview with researchers via Zoom, Facetime, or a phone call or to an in-person interview. A standardized web-based survey (http://www.surveymonkey.com (accessed on 3 March 2021)) was used by the researchers when they interviewed the participants. We restricted multiple responses from the participants by using unique identification codes for each interviewer and participant. Before we interviewed participants, a face validity test was carried out by experts in the field of oral pathology or oral medicine to check if the survey covered all the needed questions and was appropriately written. Additionally, a pilot test of the questionnaire on 20 participants was performed to study the questions’ clarity and verify reliability. We revised the survey and collection tool based on the outcomes of the pretesting procedures.

### 2.5. Data Collection

A total of 208 persons accepted our invitation and agreed to be interviewed and answer the survey, which took 10 min to complete. Survey questions asked about participants’ demographic data and health status, their history of exposure to COVID-19, symptoms of COVID-19, and oral manifestations of COVID-19. We inquired about the presence of the following general symptoms of COVID-19: fever, cough, fatigue, myalgia/arthralgia, dyspnea, headache, sore throat, nausea/vomiting, diarrhea, nasal obstruction, or loss of smell. Individuals were also free to document any other symptoms. We also obtained information on the severity of COVID-19 (i.e., mild, moderate, severe, or critical) based on the guidelines in the document titled WHO COVID-19: Clinical Management, Interim Guidelines [16]. Information on the severity of each general symptom of COVID-19, whether mild, moderate, or severe, was assessed using the subjective Visual Analogue Scale (VAS), with 0 indicating no symptom and 10 the most severe symptom [17]. We assessed the duration of each symptom (i.e., 1 to 2 days, 3 to 4 days, or 5+ days), frequency of each symptom (i.e., occurred once, occurred intermittently, or occurred constantly), whether hospitalization was needed due to COVID-19, the reason for hospitalization, and the duration of hospitalization.

To determine the prevalence of oral manifestations of COVID-19, the participants were asked about their history of xerostomia, oral ulcerations, gingivitis, necrotizing periodontal disease, candidiasis, distortion of taste, vesiculobullous lesions, erythema migrans, geographic tongue, petechiae, and leukoplakia. Participants were also free to document any other symptoms. The severity, duration, frequency, location, and outcome of the oral manifestations were documented.

### 2.6. Data Analysis

Data were downloaded from the web-based tool Survey Monkey and imported into SAS version 9.2 for analysis. Continuous variables were expressed as the mean and standard deviation if they were normally distributed or as the median and range if they were skewed. Categorical variables were expressed as frequencies and proportions. Chi-square statistics or the Fisher’s exact test was used to compare the findings and determine the proportions of participants with the findings, which were the overall severity of COVID-19, the presence or absence of oral manifestations of COVID-19, the general symptoms of COVID-19 with the presence or absence of oral manifestations of COVID-19, and the oral manifestations of COVID-19 by severity, duration, and frequency with the overall severity of COVID-19. Additionally, participants with different oral manifestations and the timing of the oral manifestations were described.

## 3. Results

### 3.1. Participant Selection

An invitation to participate in a survey was randomly sent out, and 208 individuals accepted the invitation. A total of 201 were eligible and consented to an interview by the research team. After individuals with incomplete documentation (*n* = 6) were excluded, 195 were deemed acceptable for inclusion in the final analysis.

### 3.2. Participant Characteristics, COVID-19 Disease Severity, and COVID-19 Exposure

Participants who had been previously diagnosed with COVID-19 rated their status at the time of diagnosis as asymptomatic or having mild symptoms (21%), moderate symptoms (59%), or severe and critical symptoms (20%). Of the patients, 33% were 18 to 24 years old and 33% were 25 to 34 years old. Most participants were female (75%). Approximately 33% were current or previous smokers, 18% were taking medications, and 9% had been hospitalized due to COVID-19. There was a significantly high proportion of participants older than 55 years with mild COVID-19 symptoms compared with other age groups (*p* = 0.0175). There was a significantly high proportion of moderate and severe COVID-19 symptoms in patients who were hospitalized compared with those with mild disease (*p* = 0.0002). Participants did not differ in COVID-19 severity based on gender, smoking history, or medication status (Table 1).

Patients taking medications (*n =* 36; 18%) were taking antihypertensives (7), antihistamines (8), thyroid medication (7), antidiabetics (5), antiasthmatics (3), proton pump inhibitors (2), nutritional supplements (2), antiepileptics (2), antidepressants (1), osteoarthritis medication (1), benign prostatic hyperplasia medication (1), hormone replacements (1), or laxatives (1). These categories were not mutually exclusive (data not shown).

Although most had strictly followed the recommended COVID-19 preventive measures (73%), had not travelled abroad during the pandemic (86%), and were not healthcare providers (76%), the majority had been in contact with a person with COVID-19 (82%) and half had been in crowded places or attended a group gathering (50%) (data not shown).

### 3.3. COVID-19 General Symptoms, Severity, and Hospitalization Due to COVID-19

The general COVID-19 symptoms among all participants were fever (95%, *n* = 140), headache (65%, *n* = 127), fatigue (65%, *n* = 126), cough (63%, *n* = 122), myalgia/arthralgia (53%, *n* = 104), loss of smell (53%, *n* = 102), sore throat (50%, *n* = 97), shortness of breath or dyspnea (40%, *n* = 78), nausea or vomiting (21%, *n* = 41), and diarrhea (15%, *n* = 30). These categories were not mutually exclusive; a participant could report more than one symptom. The highest proportion of patients with severe symptoms had loss of smell; this symptom lasted 5+ days and was constant throughout that entire time (Table 2). Seventeen patients (9%) were hospitalized due to COVID-19 for 1 to 21 days, 10 for 1 to 7 days, 2 for 7 to 14 days, and 2 for 15 to 21 days; the duration for three participants was not documented. The reasons for hospitalization for the 11 participants who responded to this question varied; these were allergy (one participant), precautionary (one), back pain (one), chest pain (two), difficulty in breathing (two), fever (two), diarrhea and intravenous medication (one), and headache and fever (one).

### 3.4. Oral Manifestations of COVID-19

A total of 57 participants (29%) had oral manifestations of COVID-19. The majority were age 45 to 54 years and 55+ years. There was a significantly higher proportion of females than males with oral manifestations (33% vs. 17%, *p* = 0.0275). Approximately 25% of current or previous smokers, 33% of those taking medications, and 24% of those who had been hospitalized due to COVID-19 developed oral manifestations. Thirteen participants with oral manifestations were taking medications (Table 1).

Of patients who had oral manifestations (*n* = 57, 29%), thirty-four (60%) had distortion of taste, twenty-four (42%) had xerostomia, six (11%) had oral ulcerations, three (6%) had gingivitis, three (6%) had petechiae, three (6%) had candidiasis, two (4%) had necrotizing periodontal disease, two (4%) had vesiculobullous lesions, two (4%) had erythema migrans, and two (4%) had a geographic tongue. These categories were not mutually exclusive; a participant could report more than one oral manifestation (Table 3).

### 3.5. Relationship between Oral Manifestations of COVID-19 and Overall Severity of COVID-19

Of participants with mild, moderate, severe, or critical COVID-19, 27%, 30%, 29%, and 25% of participants, respectively, developed oral manifestations. The following symptoms were described as moderate or severe and lasting 3 to 4 or 5+ days before resolution: distortion of taste, xerostomia, and oral ulcerations. Candidiasis was most often described as mild and lasting for 1 to 2 days. There was no statistically significant association between the severity, duration, and frequency of each oral manifestation of COVID-19 and the overall severity of COVID-19 symptoms (Supplementary Table S1).

### 3.6. Relationship between Oral Manifestations of COVID-19 and Specific General COVID-19 Symptoms in Terms of Severity, Duration, and Frequency

There was no relationship between the severity of specific general COVID-19 symptoms and the presence of oral manifestations. However, patients with fatigue lasting more than 5 days were more likely to develop oral manifestations (*p* < 0.0007). Patients with loss of smell for 3 to 4 days were more likely to develop oral manifestations, and the most common one was a taste disorder (*p* = 0.0122) [12]. Patients with headaches that were constant or intermittent were more likely to develop oral manifestations (*p* = 0.0336) (Table 2).

### 3.7. Timing of Oral Manifestations of COVID-19 Relative to General Symptoms of and Therapy for COVID-19

Fifty-three of the fifty-seven participants with oral manifestations could recall the time of appearance of the oral manifestations in relation to the general symptoms and therapy they received. The oral manifestations developed concurrently with the general symptoms in 47%, after the general symptoms occurred in 43%, and before the general symptoms occurred in 9%. Patients with mild disease developed oral manifestations before the appearance of general symptoms, whereas those with moderate or severe disease developed oral manifestations concurrently with or after the appearance of general symptoms (Table 4).

## 4. Discussion

COVID-19 patients presented with different symptoms and manifestations in different body systems, such as the cutaneous, gastrointestinal, and respiratory systems [8,18,19]. In this study, we aimed to determine the prevalence of oral manifestations of patients diagnosed with COVID-19 and their association with disease severity. The prevalence of oral manifestations among all participants was 29%. These patients were predominantly female, 45 to 54 years of age or older than 55 years, and nonsmokers; were not taking any medications; and had never been hospitalized for COVID-19. Patients with the oral manifestations of COVID-19 were more likely to have moderate or severe COVID-19. The most common oral manifestations of COVID-19 reported in our cohort were distortion of taste, xerostomia, and oral ulceration, which is congruent with what has been reported in the literature [9,14,20]. Martín Carreras-Presas et al. were the first to report oral ulcerations as an oral manifestation associated with COVID-19 [21]. Several reports followed a heterogeneous group of lesions in suspected and confirmed cases of COVID-19, with, as reported in the current study, altered taste and oral ulcerations being the most common symptoms [20]. The development of oral lesions in a setting of COVID-19 infection may be explained by the affinity of the coronavirus for ACE2 receptors, which are highly expressed in the respiratory and oral epithelium. The binding of the virus to these receptors may disrupt the epithelial lining and the functioning of keratinocytes [7]. However, the cause-and-effect relationship of the development of oral manifestations in confirmed cases of COVID-19 is still unclear. Whether it is a direct effect of the virus on the oral mucosal tissue or a consequence of an impaired immune response or concurrent infection needs to be investigated.

Moreover, the exacerbated vigor and activity of innate immune response mechanisms [22] and hormonal modulations in females may be responsible for the higher prevalence of oral manifestations among female participants [23], as previously reported [14,24,25]. A higher proportion of elderly patients developed oral manifestations, as in the study by Iranmanesh et al. [8]. Immune suppression due to advances in age may be responsible for a higher prevalence of oral manifestations in this age group.

Patients with oral manifestations tended to have moderate or severe COVID-19. It was observed that patients of older age and a higher degree of severity of COVID-19 suffered from more severe episodes of oral lesions [6,8,10]. Oral manifestations developed regardless of the severity of COVID-19. However, some studies found that most of the oral manifestations were linked to severe COVID-19 [26,27]. This could be attributed to the hyperinflammatory response to COVID-19 [8]. Nonetheless, we were able to establish a link between the presence of oral manifestations and the presence of specific general symptoms of COVID-19. Similar to other cohorts studied in Saudi Arabia, most of the participants in our study described the general symptom of a chemosensory disorder, such as the loss of smell and taste, as being severe and extending for more than 5 days [28]. Loss of taste along with anosmia was concurrent and attributed to edema of the respiratory system [6]. This lends credence to the theory that olfactory and gustatory dysfunctions are potential indications of COVID-19. Xerostomia was the second most common manifestation (41%; *n =* 24) in the current study in patients who were nonsmokers and were not taking medications; it appeared concurrently with general symptoms and was associated with moderate severity of COVID-19. Another study showed that xerostomia was the most common oral manifestation in COVID-19 patients [23], indicating that salivary gland symptoms and disorders are highly prevalent among these patients. However, further case–control studies are suggested to confirm this observation.

Patients with mild disease developed oral manifestations prior to the onset of general symptoms, whereas those with more severe disease developed oral manifestations at the same time or after the appearance of general symptoms. Oral symptoms could have developed secondarily to the use of medications or to adverse reactions to medications such as corticosteroids with increased severity of the disease. Other studies reported the appearance of oral lesions after the onset of COVID-19 regardless of the severity of disease [24].

There are some limitations in the current study, which was conducted to be descriptive only, without multivariant analysis. It was not possible to analyze the oral manifestations for some patients because the data regarding duration and frequency of their self-reported symptoms were incomplete. Because the study relied on participants’ self-reporting, the prevalence of some symptoms may have been overestimated or underestimated. Moreover, the current study cannot be generalized, because the validity of the self-reported symptoms was not corroborated by objective measures, and therefore reporting bias could not be prevented. We excluded patients under age 18 years, so our study did not provide data for this population. To our knowledge, our study was the first study that addressed the relationship between oral manifestations of COVID-19 and specific general symptoms of COVID-19 in terms of severity, duration, and frequency. This study reported the oral manifestations associated with COVID-19 in patients with symptoms ranging from mild to severe. We found that taste distortion could increase the clinical suspicion for COVID-19 and could be a potential indicator for patient screening.

Oral manifestations of COVID-19 were common in female patients and were linked to specific general COVID-19 symptoms. Taste distortion, xerostomia, and oral ulcerations were the most reported oral manifestations. Future studies should be undertaken to elucidate the controversial link between oral lesions and COVID-19 infection using validated measurement tools. Dentists and other health practitioners should be careful during dental examinations because oral manifestations of COVID-19 may emerge before the onset of general symptoms of COVID-19, as they did in this study population. Dental professionals may also promote awareness of the common oral manifestations of COVID-19, which may persist for long durations, and provide the proper management and medical attention for them.

## Figures and Tables

**Table 1:** Participant characteristics of COVID-19 with and without oral manifestations.

Participant Characteristics	All Participants (*N =* 195) *n*/*N*	COVID-19 Severity			*p*-Value **	Oral Manifestations of COVID-19		*p*-Value **
		Mild 41 (21%)	Moderate 115 (59%)	Severe and Critical ** 39 (20%)		Had Oral Manifestations of COVID-19 (*n* = 57; 29%)	Did Not Have Oral Manifestations of COVID-19 (*n* = 138; 71%)	
Age group:								
18–24 years	64 (33%)	15 (23%)	41 (64%)	8 (13%)	**0.0175**	18 (28%)	46 (72%)	0.3166
25–34 years	64 (33%)	7 (11%)	43 (67%)	14 (22%)		18 (285)	46 (72%)	
35–44 years	41 (21%)	12 (29%)	22 (54%)	7 (17%)		9 (22%)	32 (78%)	
45–54 years	13 (7%)	2 (15%)	5 (39%)	6 (46%)		6 (46%)	7 (54%)	
55+ years	13 (5%)	5 (38%)	4 (31%)	4 (31%)		6 (46%)	7 (54%)	
Sex:								
Female	147 (75%)	27 (18%)	88 (60%)	32 (22%)	0.2208	49 (33%)	98 (67%)	**0.0275**
Male	48 (25%)	14 (29%)	27 (56%)	7 (15%)		8 (17%)	40 (83%)	
Smoking history (current and previous smoker)	65 (33%)	9 (14%)	39 (60%)	17 (26%)	0.1192	16 (25%)	43 (75%)	0.3163
Taking medication ^€^	36 (18%)	9 (25%)	21 (58%)	9 (17%)	0.7482	49 (33%)	98 (67%)	0.0275
Positive history of hospitalization due to COVID-19	17 (9%)	0 (0%)	7 (41%)	10 (59%)	**0.0002**	4 (24%)	13 (76%)	0.7819

** Chi = square statistics or Fisher’s exact test. ^€^ These categories were not mutually exclusive.

**Table 2:** Relationships between general symptoms of COVID-19 in terms of severity, duration, and the frequency and presence of oral manifestations of COVID-19.

Symptom	Total 195	Severity of Specific COVID-19 Symptom				Duration of Specific COVID-19 Symptom				Frequency of Specific COVID-19 Symptom			
		Mild	Moderate	Severe	*p*-Value	1–2 Days	3–4 Days	5+ Days	*p*-Value	Once	Intermittent	Constant	*p*-Value
Fever	140 (72%)	34 (24%)	81 (58%)	25 (18%)		47 (35%)	46 (35%)	40 (30%)		13 (11%)	65 (53%)	45 (36%)	
*Oral manifestations +*	43 (31%)	9 (26%)	30 (37%)	4(16%)	0.8751	15 (32%)	13 (28%)	14 (35%)	0.7971	5 (39%)	22 (34%)	11 (24%)	0.4745
*Oral manifestations −*	97 (69%)	25 (74%)	51 (68%)	21 (84%)		32 (68%)	33 (72%)	26 (65%)		8 (62%)	43 (66%)	34 (76%)	
Headache	127 (65%)	33 (24%)	42 (33%)	52 (41%)		25 (22%)	29 (25%)	61 (53%)		10 (9%)	55 (49%)	47 (42%)	
*Oral manifestations +*	38 (29%)	11 (33%)	12 (29%)	15 (28%)	0.8832	6 (24%)	9 (31%)	20 (33%)	0.7214	0 (0%)	22 (40%)	22 (40%)	0.0336
*Oral manifestations −*	89 (71%)	22 (67%)	30 (71%)	37 (72%)		19 (76%)	20 (69%)	41 (67%)		10 (100%)	33 (60%)	34 (72%)	
Fatigue	126 (65%)	33 (26%)	48 (38%)	45 (36%)		19 (16%)	36 (31%)	61 (53%)		3 (3%)	37 (34%)	69 (63%)	
*Oral manifestations +*	40 (32%)	13 (39%)	17 (35%)	10 (22%)	0.2151	2 (11%)	7 (19%)	30 (49%)	0.0007	0 (0%)	13 (35%)	22 (32%)	0.4548
*Oral manifestations −*	86 (68%)	20 (61%)	31 (65%)	35 (78%)		17 (89%)	29 (82%)	31 (51%)		3 (100%)	24 (65%)	47 (68%)	
Cough	122 (63%)	41 (34%)	49 (40%)	32 (26%)		19 (17%)	29 (25%)	66 (58%)		5 (5%)	56 (55%)	41 (40%)	
*Oral manifestations +*	39 (32%)	17 (42%)	16 (33%)	6 (19%)	0.1176	3 (16%)	12 (41%)	23 (35%)	0.1669	2 (40%)	21 (38%)	11 (27%)	0.5188
*Oral manifestations −*	83 (68%)	24 (58%)	33 (67%)	26 (81%)		16 (84%)	17 (59%)	43 (65%)		3 (60%)	35 (62%)	30 (73%)	
Myalgia/arthralgia	104 (53%)	26 (25%)	37 (36%)	41 (39%)		18 (19%)	24 (26%)	52 (55%)		6 (54%)	36 (40%)	49 (6%)	
*Oral manifestations +*	32 (31%)	11 (42%)	13 (35%)	8 (20%)	0.1111	3 (17%)	8 (33%)	20 (38%)	0.2375	0 (0%)	16 (44%)	15 (31%)	0.0736
*Oral manifestations −*	72 (69%)	15 (58%)	24 (65%)	33 (80%)		15 (83%)	16 (67%)	32 (62%)		6 (100%)	20 (56%)	34 (69%)	
Loss of smell	102 (53%)	9 (9%)	19 (19%)	74 (72%)		5 (5%)	8 (9%)	80 (86%)		1 (1%)	4 (5%)	78 (94%)	
*Oral manifestations +*	40 (39%)	5 (55%)	10 (53%)	25 (34%)	0.1865	1 (20%)	7 (88%)	31 (39%)	0.0122	0 (0%)	2 (50%)	33 (42%)	1.0000
*Oral manifestations −*	62 (61%)	4 (44%)	9 (47%)	49 (66%)		4 (80%)	1 (12%)	49 (61%)		1 (100%)	2 (50%)	45 (58%)	
Sore throat	97 (50%)	33 (34%)	38 (39%)	26 (27%)		18 (20%)	39 (44%)	32 (36%)		9 (11%)	27 (33%)	47 (56%)	
*Oral manifestations +*	34 (35%)	14 (42%)	10 (26%)	10 (38%)	0.3338	5 (28%)	15 (38%)	12 (38%)	0.7181	2 (22%)	11 (41%)	17 (36%)	0.6056
*Oral manifestations −*	63 (65%)	19 (58%)	28 (74%)	16 (62%)		13 (72%)	24 (62%)	20 (62%)		7 (78%)	26 (59%)	30 (64%)	
Dyspnea	78 (40%)	22 (28%)	24 (31%)	32 (41%)		10 (15%)	22 (32%)	36 (53%)		8 (12%)	35 (54%)	22 (34%)	
*Oral manifestations +*	22 (28%)	8 (36%)	7 (29%)	7(22%)	0.5048	2 (20%)	8 (36%)	11 (31%)	0.6485	2 (25%)	13 (37%)	5 (23%)	0.4818
*Oral manifestations −*	56 (72%)	14 (64%)	17 (71%)	25 (78%)		8 (80%)	14 (64%)	25 (69%)		6 (75%)	22 (67%)	17 (77%)	
Nausea/ vomiting	41 (21%)	20 (49%)	15 (36%)	6 (15%)		11 (34%)	7 (22%)	14 (44%)		5 (13%)	22 (71%)	4 (16%)	
*Oral manifestations +*	11 (27%)	5 (25%)	5 (33%)	1 (17%)	0.7991	1 (9%)	3 (43%)	5 (36%)	0.2099	2 (40%)	6 (27%)	1 (25%)	0.8367
*Oral manifestations −*	30 (73%)	15 (75%)	10 (67%)	5 (83%)		10 (91%)	4 (57%)	9 (64%)		3 (60%)	16 (73%)	3 (75%)	
Diarrhea	30 (15%)	19 (63%)	8 (27%)	3 (10%)		13 (57%)	4 (17%)	6 (26%)		6 (29%)	11 (52%)	4 (19%)	
*Oral manifestations +*	11 (37%)	7 (37%)	4 (50%)	0 (0%)	0.3861	6 (46%)	1 (25%)	2 (33%)	0.7085	4 (67%)	3 (27%)	1 (25%)	0.2329
*Oral manifestations −*	19 (63%)	12 (63%)	4 (50%)	3 (100%)		7 (54%)	3 (75%)	4 (67%)		2 (33%)	8 (73%)	3 (75%)	

Oral manifestations +: Patient(s) developed oral manifestations of COVID-19 simultaneously with having the general symptom listed just above it. Oral manifestations −: Patient(s) did not have oral manifestations of COVID-19 simultaneously with the general symptom listed above it.

**Table 3:** Characteristics of patients with oral manifestations of COVID-19.

Participant Characteristics	All Participants with Oral Manifestations of COVID-19 *N =* 57 *n*/*N*	Oral Manifestation of COVID-19 among 195 Participants									
		Dysgeusia (*n* = 34; 60%)	Xerostomia (*n* = 24; 42%)	Oral Ulceration (*n* = 6; 11%)	Gingivitis (*n* = 3; 6%)	Petechiae (*n* = 3; 6%)	Candidiasis (*n* = 3; 6%)	Necrotizing Periodontal Disease (*n =* 2; 4%)	Vesiculobullous Lesions (*n =* 2; 4%)	Erythema Migrans (*n* = 2; 4%)	Geographic Tongue (*n* = 2; 4%)
Age group											
18–24 years	18 (32%)	11 (32%)	5 (21%)	4 (67%)	1 (33%)	1 (33%)	1 (50%)	1 (50%)	1 (50%)	1 (50%)	1 (50%)
25–34 years	18 (32%)	13 (38%)	8 (33%)	1 (17%)	1 (33%)	2 (67%)	0 (0%)	1 (50%)	1 (50%)	1 (505)	1 (50%)
35–44 years	9 (16%)	6 (18%)	5 (13%)	0 (0%)	0 (0%)	0 (0%)	1 (50%)	0 (0%)	0 (0%)	0 (0%)	0 (0%)
45–54 years	6 (10%)	3 (95%)	5 (21%)	1 (17%)	0 (0%)	0 (0%)	0 (0%)	0 (0%)	0 (0%)	0 (0%)	0 (0%)
55+ years	6 (10%)	1 (3%)	3 (12%)	0 (0%)	1 (33%)	0 (0%)	1 (6%)	0 (0%)	0 (0%)	0 (0%)	0 (0%)
Sex (female)	49 (86%)	30 (88%)	21 (87%)	5 (83%)	2 (67%)	2 (67%)	2 (67%)	1 (50%)	1 (50%)	1 (50%)	1 (50%)
Smoking history (current or previous smoker)	16 (28%)	9 (27%)	7 (29%)	3 (50%)	1 (33%)	2 (67%)	0 (0%)	1 (50%)	1 (50%)	1 (50%)	1 (50%)
Taking medication ^€^	13 (23%)	7 (21%)	4 (17%)	0 (0%)	1 (33%)	0 (0%)	2 (15%)	0 (0%)	0 (0%)	0 (0%)	0 (0%)
Positive history of hospitalization	4 (7%)	1 (3%)	1 (4%)	1 (17%)	0 (0%)	0 (0%)	1 (33%)	0 (0%)	0 (0%)	0 (0%)	0 (0%)

^€^ These categories are not mutually exclusive.

**Table 4:** Temporal relationship of oral manifestations of and general symptoms of COVID-19 in 53 of the 57 participants with oral manifestations of COVID-19.

COVID-19 Severity	Total ^±^ 57 *n*/*N*	Timing of Oral Manifestations Relative to COVID-19 General Symptoms		
		Before COVID-19 General Symptoms (*n =* 5; 9%)	Concurrent with COVID-19 General Symptoms (*n =* 25; 47%)	After COVID-19 General Symptoms (*n =* 23; 43%)
Mild	11 (19%)	3 (60%)	4 (16%)	4 (17%)
Moderate	35 (61%)	2 (40%)	15 (60%)	15 (65%)
Severe or critical	11 (19%)	1 (20%)	6 (24%)	4 (17%)

^±^ Three patients with distortion of taste did not give information on the timing of oral manifestations relative to COVID-19 general symptoms.

## Data Availability

The data presented in this study are available on request from the corresponding author.

## Supplementary Materials

The following supporting information can be downloaded at: https://www.mdpi.com/article/10.3390/jcm11154461/s1, Table S1: Frequency, severity, and duration of oral manifestations of COVID-19.
