# Supplementary material for: Oral Manifestations of COVID-19: A Cross-Sectional Study of Their Prevalence and Association with Disease Severity
Source: J Clin Med. 2022 Jul 30;11(15):4461. doi: 10.3390/jcm11154461 (PMC9369841; doi:10.3390/jcm11154461)
Supplement: Supplementary file 1 [file jcm-11-04461-s001.zip › jcm-1824278-supplementary.pdf]

Supplementary Table S1. Frequency, severity, and duration of oral manifestations of COVID-19.

**Symptoms**		**All participants N=57 n/N**	**Mild COVID-19 disease symptoms 11 (19)**	**Moderate COVID-19 disease symptoms 35 (62)**	**Severe & Critical COVID-19 disease symptoms π 11 (19)**	**P value π π**
Distortion of taste *						
Total		34 (60)	7 (21)	24 (70)	3 (9)	0.0658
Severity	Mild	5 (15)	2 (40)	3 (60)	0 (0)	0.5687
	Moderate	11 (33)	2 (18)	7 (64)	2 (18)	
	Severe	**18 (53)**	3 (17)	14 (78)	1 (5)	
Duration	1-2 days	0 (0)	0 (0)	0 (0)	0 (0)	0.7873
	3-4 days	6 (18)	2 (33)	4 (67)	0 (0)	
	5+ days	**27 (82)**	5 (19)	19 (70)	3 (11)	
Xerostomia**						
Total		24 (42)	5 (21)	12 (50)	7 (64)	0.2857
Severity	Mild	5 (21)	2 (40)	3 (60)	0 (0)	0.3919
	Moderate	**16 (67)**	2 (12)	8 (50)	6 (38)	
	Severe	3 (12)	1 (33)	1 (33)	1 (33)	
Duration	1-2 days	3 (13)	0 (0)	3 (13)	0 (0)	0.3453
	3-4 days	9 (39)	1 (11)	4 (44)	4 (44)	
	5+ days	**11 (47)**	4 (36)	4 (36)	3 (37)	
Oral ulceration						
Total		6 (11)	0 (0)	4 (67)	2 (33)	0.4104
Severity	Mild	1 (17)	0 (0)	1 (100)	0 (0)	
	Moderate	**4 (67)**	0 (0)	3 (75)	1 (25)	
	Severe	1 (16)	0 (0)	0 (0)	1 (100)	
Duration	1-2 days	0 (0)	0 (0)	0 (0)	0 (0)	-
	3-4 days	2 (33)	0 (0)	2 (100)	0 (0)	
	5+ days	**4 (67)**	0 (0)	2 (50)	2 (50)	
Gingivitis ***						
Total		3 (6)	0 (0)	3 (100)	0 (0)	-
Severity	Mild	**3 (100)**	0 (0)	3 (100)	0 (0)	-
	Moderate	0 (0)	0 (0)	0 (0)	0 (0)	
	Severe	0 (0)	0 (0)	0 (0)	0 (0)	

Duration	1-2 days	0 (0)	0 (0)	0 (0)	0 (0)	-
	3-4 days	0 (0)	0 (0)	0 (0)	0 (0)	
	5+ days	0 (0)	0 (0)	0 (0)	0 (0)	
Petechiae[+]						
Total		3 (6)	0 (0)	3 (100)	0 (0)	-
Severity	Mild	**2 (67)**	0 (0)	2 (100)	0 (0)	-
	Moderate	1 (33)	0 (0)	1 (100)	0 (0)	
	Severe	0 (0)	0 (0)	0 (0)	0 (0)	
Duration	1-2 days	0 (0)	0 (0)	0 (0)	0 (0)	-
	3-4 days	0 (0)	0 (0)	0 (0)	0 (0)	
	5+ days	1 (100)	0 (0)	1 (100)	0 (0)	
Candidiasis						
Total		3 (6)	1 (33)	1 (33)	1 (33)	0.3289
Severity	Mild	**2 (67)**	1 (50)	1 (50)	0 (0)	1.0000
	Moderate	0 (0)	0 (0)	0 (0)	0 (0)	
	Severe	1 (33)	0 (0)	0 (0)	1 (100)	
Duration	1-2 days	**5 (83)**	3 (60)	2 (40)	0 (0)	0.1667
	3-4 days	0 (0)	0 (0)	0 (0)	0 (0)	
	5+ days	1 (17)	0 (0)	0 (0)	1 (100)	
Necrotizing periodontal disease[¥]						
Total		2 (4)	0 (0)	2 (100)	0 (0)	1.0000
Severity	Mild	**2 (100)**	0 (0)	2 (100)	0 (0)	-
	Moderate	0 (0)	0 (0)	0 (0)	0 (0)	
	Severe	0 (0)	0 (0)	0 (0)	0 (0)	
Duration	1-2 days	0 (0)	0 (0)	0 (0)	0 (0)	-
	3-4 days	0 (0)	0 (0)	0 (0)	0 (0)	
	5+ days	0 (0)	0 (0)	0 (0)	0 (0)	
Vesiculobullous lesions[±]						
Total		2 (4)	0 (0)	2 (100)	0 (0)	1.000
Severity	Mild	**2 (100)**	0 (0)	2 (100)	0 (0)	-
	Moderate	0 (0)	0 (0)	0 (0)	0 (0)	
	Severe	0 (0)	0 (0)	0 (0)	0 (0)	
Duration	1-2 days	0 (0)	0 (0)	0 (0)	0 (0)	-
	3-4 days	0 (0)	0 (0)	0 (0)	0 (0)	
	5+ days	0 (0)	0 (0)	0 (0)	0 (0)	

Erythema migrans [±±]						
Total		2 (4)	0 (0)	2 (100)	0 (0)	1.000
Severity	Mild	**2 (100)**	0 (0)	2 (100)	0 (0)	-
	Moderate	0 (0)	0 (0)	0 (0)	0 (0)	
	Severe	0 (0)	0 (0)	0 (0)	0 (0)	
Duration	1-2 days	0 (0)	0 (0)	0 (0)	0 (0)	-
	3-4 days	0 (0)	0 (0)	0 (0)	0 (0)	
	5+ days	0 (0)	0 (0)	0 (0)	0 (0)	
Geographical tongue [β]						
Total		2 (4)	0 (0)	2 (100)	0 (0)	1.000
Severity	Mild	**2 (100)**	0 (0)	2 (100)	0 (0)	-
	Moderate	0 (0)	0 (0)	0 (0)	0 (0)	
	Severe	0 (0)	0 (0)	0 (0)	0 (0)	
Duration	1-2 days	0 (0)	0 (0)	0 (0)	0 (0)	-
	3-4 days	0 (0)	0 (0)	0 (0)	0 (0)	
	5+ days	0 (0)	0 (0)	0 (0)	0 (0)	

[π] 1 of those with severe and critical symptoms had critical symptoms of COVID-19 disease

[π π] Chi square or fisher's test

* 1 missing information on dysgeusia duration

** 1 missing information on xerostomia duration

*** 3 missing information on gingivitis duration

[+]2 missing information on petechiae duration

[ɣ] information not collected on duration of necrotizing periodontal disease

[±] 2 missing information on vesicobullous disease duration

[±±] 2 missing information on Erythema migrans duration

[β] 2 missing information on geographical tongue duration
